# Disturbances of the Gut Microbiota, Sleep Architecture, and mTOR Signaling Pathway in Patients with Severe Obstructive Sleep Apnea-Associated Hypertension

**DOI:** 10.1155/2021/9877053

**Published:** 2021-11-30

**Authors:** Chih-Yuan Ko, Huan-Zhang Su, Li Zhang, Yi-Ming Zeng

**Affiliations:** ^1^Department of Respiratory and Critical Care Medicine, The Second Affiliated Hospital of Fujian Medical University, Quanzhou 362000, China; ^2^Department of Clinical Nutrition, The Second Affiliated Hospital of Fujian Medical University, Quanzhou 362000, China; ^3^School of Public Health, Fujian Medical University, Fuzhou 350122, Fujian, China; ^4^Respiratory Medicine Center of Fujian Province, Quanzhou 362000, China; ^5^Department of Respiratory, The First Hospital of Quanzhou, Quanzhou 362000, China

## Abstract

Intermittent hypoxia and sleep fragmentation are pathophysiological processes involved in obstructive sleep apnea (OSA) which affect gut microbiota, sleep architecture, and mTOR signaling pathway. However, the involvement of these elements in the pathogenesis mechanism of OSA-associated hypertension remains unclear. Therefore, this study investigated whether the OSA-associated hypertension mechanism is regulated by the gut microbiota and mTOR signaling pathway. Patients were diagnosed by polysomnography; their fecal samples were obtained and analyzed for their microbiome composition by 16S ribosomal RNA pyrosequencing and bioinformatics analysis. Transcript genes on fasting peripheral blood mononuclear cells (PBMCs) were examined using Illumina RNA-sequencing analysis. Totally, we enrolled 60 patients with severe OSA [without hypertension (*n* = 27) and with hypertension (*n* = 33)] and 12 controls (neither OSA nor hypertension). Results revealed that severe-OSA patients with hypertension had an altered gut microbiome, decreased short-chain fatty acid-producing bacteria (*P* < 0.05), and reduced arginine and proline metabolism pathways (*P*=0.001), compared with controls; also, they had increased stage N1 sleep and reduced stages N2 and N3 sleep accompanied by repeated arousals (*P* < 0.05). Analysis of PBMCs using the Kyoto Encyclopedia of Genes and Genomes database showed that the mTOR signaling pathway (*P*=0.006) was the most important differential gene-enriched pathway in severe-OSA patients with hypertension. Our findings extend prior work and suggest a possibility that the regulation of the mTOR signaling pathway is involved in developing OSA-associated hypertension through its interaction with the disturbance of the gut microbiome and sleep architecture.

## 1. Introduction

Obstructive sleep apnea (OSA) is a common clinical sleep disorder affecting the entire body, and it is related to the increased morbidity and mortality of cardiovascular and cerebrovascular diseases [1]. Intermittent hypoxia (IH) and sleep fragmentation (SF) are important hallmarks of OSA which are partially attributed to decreased airflow (hypopnea) or interrupted breathing (apnea), accompanied by decreased blood oxygen saturation and hypercapnia. These pathophysiological processes can lead to a series of complications, including hypertension, cerebrovascular events, or metabolic disorders [2].

OSA is an independent risk factor for hypertension. The severity of hypertension positively correlates with the apnea-hypopnea index (AHI). Continuous positive airway pressure (CPAP) treatment can substantially reduce the blood pressure of patients with OSA and hypertension (OSA-HTN), especially those with refractory hypertension [3, 4]. IH can lead to decreased oxygen saturation, increased vascular pressure, excessive activation of sympathetic nerve activity, autonomic dysfunction, and apnea that awakens the central nervous system. It can ultimately lead to vascular endothelial dysfunction and multiple organ damage. Its underlying mechanism involves a cascade of inflammation and oxidative stress [5]. CPAP treatment has a protective effect on the neurocognition of patients with OSA whether gut microbiota is involved in the neurocognitive physiological mechanism of OSA unclearly [6]. Recent research has shown that gut microbiota plays a critical role in regulating the risk of chronic diseases and maintaining intestinal immunity and systemic homeostasis. They significantly affect the onset of obesity, cardiometabolic abnormalities, and mental illness [7]. Interestingly, these lesions can be seen in patients with OSA.

SF can cause vital organ disruptions independent of IH. SF is with repeated arousals implicated in overactivation of sympathetic neural activity. The principal mechanism of OSA-induced brain damage involves disrupting different stages of sleep by causing rapid eye movement (REM) and non-REM (NREM) sleep interruptions [8]. Studies have found that sleep architecture changes and reduced deep sleep time may lead to increased blood pressure, indicating that SF may have a certain mechanism in OSA-associated hypertension [9–11]. Moreover, SF promotes obesity and metabolic abnormalities, mediated by changes in the host intestinal microbiota and systemic adipose tissue inflammation accompanied by insulin resistance [12, 13].

Animal models mimicking IH and SF in OSA have demonstrated that the gut microbiota and their metabolites, such as bile acids and short-chain fatty acid (SCFAs), were altered [12, 14–16]. Our previous human study revealed that the gut microbiota is unbalanced. The microbiome's metabolic function analysis shows changes also combined with elevated proinflammatory cytokines in patients with different OSA severity levels [17]. Additionally, we found that the sleep architecture is disrupted, especially in patients with an AHI ≥ 15 who have the *Prevotella* enterotype [18]. The gut microbiota may be involved in regulating the structure of sleep and blood pressure through the brain-gut axis [19]. Therefore, the correlations between the gut microbiome's role in the development of OSA itself and its complications are worth investigating.

However, the mechanism of OSA-associated hypertension remains unclear. Animal experiments with rodents have confirmed that the gut microbiotal composition can be altered by regulating the mechanistic target of rapamycin (mTOR) signaling pathway under such conditions as hypoxia and energy deficiency [20, 21]. Furthermore, the correlation between gut microbiome alteration and blood pressure affects the development of hypertension [22, 23]. mTOR is a highly conserved serine/threonine-protein kinase whose signaling pathway is involved in the regulation of oxidative stress, immunity, metabolic disorders, and inflammation [24–28]. Moreover, probiotics regulating immune balance via the mTOR signaling pathway are effective in treating hypertension [23].

We hypothesized that the changes in the gut microbiota and mTOR signaling pathway in patients with OSA are involved in developing OSA-associated hypertension. Thus, we investigated whether the gut microbiota regulates the mechanism of OSA-associated hypertension, sleep architecture, and mTOR signaling pathway by evaluating the gut microbiota, polysomnography (PSG) data, and mTOR levels of peripheral blood mononuclear cells (PBMCs) of severe-OSA patients with hypertension and those without hypertension.

## 2. Materials and Methods

### 2.1. Subjects

The Institutional Review Board of the Second Affiliated Hospital of Fujian Medical University (Quanzhou, China) approved this study (approval no. 2017–78). Patients with unexplained diarrhea, infection, or gastrointestinal diseases and who used antibiotics or probiotics approximately 1 month before recruitment began were excluded. In total, 72 subjects were recruited. They underwent a full night of PSG (SOMNOscreen plus; SOMNOmedics, Randersacker, Germany) conducted by technologists in a sleep laboratory. Fasting blood or fecal samples were collected on the following morning.

### 2.2. OSA Assessments

OSA was evaluated as previously described [29]. All the patients underwent PSG with a computerized polysomnographic system, which covered electro-oculography, electroencephalography, electromyography, electrocardiography, and dynamic blood pressure measurements, including systolic blood pressure (SBP) and diastolic blood pressure (DBP). High blood pressure was defined as SBP ≥140 mmHg and/or DBP ≥90 mmHg or determined based on the patients' self-reported use of medications for hypertension. After the examination, AHI obtained the total number of apnea episodes (continuous cessation of airflow for at least 10 s) and hypopnea episodes (a 30% drop in the nasal pressure excursion for ≥10 seconds with ≥3% oxygen desaturation or arousal) by dividing the total sleep time to calculate the number of hypopneas plus apneas per hour during sleep, according to the diagnostic criteria of the 2012 American Academy of Sleep Medicine. Patients with an AHI ≤5 were assigned to the non-OSA control group, whereas patients with an AHI ≥30 were classified as the severe-OSA group.

### 2.3. Sampling, DNA Extraction, and 16S rRNA Gene Amplification Sequencing, Bioinformatic, Predictive Function

All fresh fecal samples were collected and stored in a Microbiome Test Kit (G-BIO Biotech, Hangzhou, China). 16S ribosomal RNA sequencing was conducted as described in our previous study [17]. The total sequence data of the fecal samples were analyzed for microbiome taxa based on the Quantitative Insights into Microbial Ecology bioinformatics pipeline for performing taxonomy assignments using the operational taxonomic unit method. The Phylogenetic Investigation of Communities by Reconstruction of Unobserved States bioinformatics software package and Kyoto Encyclopedia of Genes and Genomes (KEGG) database were used to predict bacterial metabolic functions.

### 2.4. Establishment of the Individuals' Transcriptome of PBMCs for RNA Sequencing

In total, 5 mL of venous blood was collected from 6 non-OSA subjects with an AHI ≤5 without hypertension and 6 OSA-HTN patients with an AHI ≥30. The complementary DNA was reverse-transcribed from the total RNA extracted from PBMCs and prepared according to reported protocols [30]. According to the manufacturer's instructions, RNA quality was analyzed using the Agilent Bioanalyzer system (Agilent, USA). RNA-sequencing assay was carried out on the Illumina HiSeq 2000 platform (Illumina, San Diego, CA, USA), and 150 base pairs of paired-end reads were obtained. A complementary DNA library was established, and sequencing was performed according to the Illumina standard protocol published by Novogene (https://en.novogene.com).

The FastQC software was used for raw read count data from the quality control of RNA-sequencing libraries. Clean reads were obtained by evaluating the QC and A/T/G/C content and filtering the raw reads. The specific criteria were as follows: (1) removal of reads with a connector (adapter); (2) removal of reads with N with a percentage greater than 10%; (3) removal of low-quality reads (reads in which the number of bases with quality value Qphred ≤20 accounts for more than 50% of the whole read). All downstream analyses were based on the clean data with high quality. Bioinformatics software analysis was performed with the R package DESeq using sequencing results for reference sequence comparison, expression statistics, and differential gene screening. Genes with a *Q* value of <0.05 were considered differentially expressed and retained for further analysis. Cluster analysis of differentially expressed genes was applied to describe their expression patterns under different treatments. KEGG enrichment analysis of the biological processes and molecular functions of up- and downregulated differentially expressed genes between control patients without OSA and patients with OSA-HTN was performed via Enrichr. The *p*-value of enrichment analysis has been adjusted in false discovery rate and was computed using the Fisher exact test.

### 2.5. Statistical Analyses

Differences in fecal microbiota were analyzed and performed by the Kruskal–Wallis test and R statistics, as appropriate. Further data analysis by *t*-test or one-way analysis of variance (ANOVA) was undertaken with SPSS version 19.0 (SPSS Inc., Chicago, IL, USA), and the data are expressed as the mean ± standard deviation (SD). Significant differences within groups were analyzed with ANOVA, followed by post hoc Fisher's least significant difference corrections for multiple normally distributed variance data comparisons. We considered a two-sided *P* < 0.05 to be statistically significant.

## 3. Results

### 3.1. Participant Characteristics

After PSG assessment, we recruited a total of 60 patients with severe OSA (without hypertension, *n* = 27; with hypertension, *n* = 33) and 12 non-OSA without hypertension control subjects (AHI of ≤5). Weight (*P* < 0.001), body mass index (*P* < 0.001), waist circumference (*P* < 0.001), and hip circumference (*P*=0.002) in the OSA group were significantly greater than those in the non-OSA group (Table 1).

### 3.2. Alterations in Taxa among Groups

**Figure 1:**
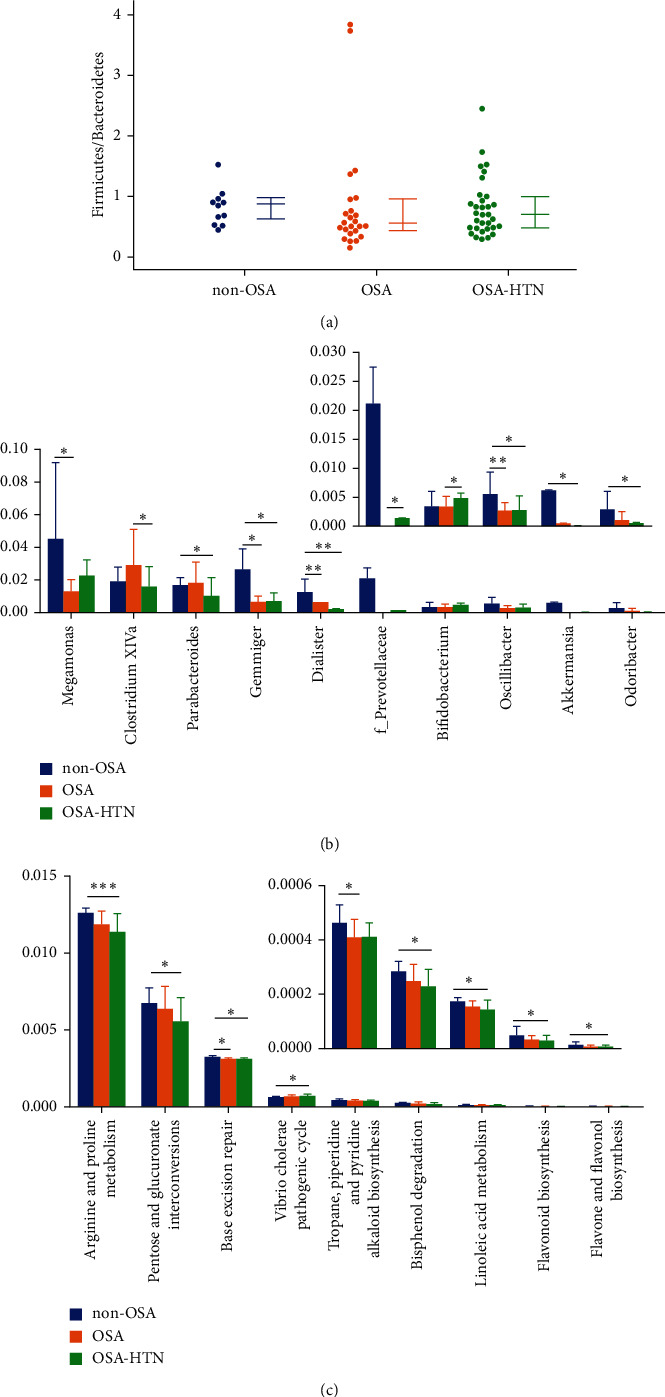
Alteration of the gut microbiome in severe obstructive sleep apnea (OSA) patients with hypertension and those without hypertension. (a) The *Firmicutes* to *Bacteroidetes* ratio is similar across groups at the phylum level. (b) The Kruskal–Wallis test was used to analyze differences in the relative abundance of the fecal microbiota at the genera level. (c) Predictive functional analysis revealed that the fecal microbiome exhibited significant Kyoto Encyclopedia of Genes and Genomes pathways. Statistical analysis was performed with the Kruskal–Wallis test. Non-OSA: apnea-hypopnea index (AHI)≤5 without hypertension, OSA: severe OSA (AHI ≥ 30) without hypertension, and OSA-HTN: severe OSA with hypertension. ^*∗*^*P* < 0.05, ^*∗∗*^*P* < 0.01, and ^*∗∗∗*^*P* < 0.001 compared with the non-OSA group or the OSA group.

We examined the mean community diversity indices (Supplementary Figure 1S). At the phylum level, we found no significant differences in the F/B ratio (Figure 1(a)). The relative abundance of *Megamonas*(*P*=0.034), *Gemmiger*(*P*=0.012), *Dialister*(*P*=0.002), and *Oscillibacter*(*P*=0.006) genera in the non-OSA group was significantly higher than in the OSA group. The relative abundance of *Parabacteroides*(*P*=0.027), *Gemmiger*(*P*=0.026), *Dialister*(*P*=0.008), *Oscillibacter*(*P*=0.019), *Akkermansia*(*P*=0.041), and *Odoribacter*(*P*=0.019) genera in the non-OSA group was significantly higher than those in the OSA-HTN group. *Clostridium* XlVa (*P*=0.046) was in the OSA group significantly higher than in the OSA-HTN group. Conversely, f__Prevotellaceae (*P*=0.030) and *Bifidobacterium*(*P*=0.021) genera were in the OSA group significantly lower than in the OSA-HTN group (Figure 1(b)).

### 3.3. Predictive Functional Analysis

Based on the KEGG database, significant differences in the fecal microbiome between the study groups were detected. Pathways of base excision repair (*P*=0.027) as well as tropane, piperidine, and pyridine alkaloid biosynthesis (*P*=0.041) were significantly higher than in the OSA group. Pathways of arginine and proline metabolism (*P*=0.001), pentose and glucuronate interconversions (*P*=0.048), base excision repair (*P*=0.026), the *Vibrio cholerae* pathogenic cycle (*P*=0.018), bisphenol degradation (*P*=0.040), linoleic acid metabolism (*P*=0.036), and flavonoid biosynthesis (*P*=0.025), as well as flavone and flavonol biosynthesis (*P*=0.045), in the non-OSA group were significantly higher than those in the OSA-HTN group (Figure 1(c)).

### 3.4. PSG Parameter Analysis

N1 sleep stage, arousal index in NREM, total sleep arousal index, apnea-hypopnea index, obstructive apnea index, central apnea index, mixed apnea index, hypopnea index, oxygen desaturation index, blood pressure elevation index, and the highest systolic blood pressure in the non-OSA group were significantly lower than the OSA group and the OSA-HTN group (Table 2). On the contrary, N2 sleep stage, N3 sleep stage, lowest oxygen saturation, and average oxygen saturation in the non-OSA group were significantly higher than the OSA group and the OSA-HTN group (Table 2). The OSA-HTN group had the highest average systolic blood pressure and average diastolic blood pressure among three groups (Table 2).

### 3.5. RNA-Sequencing Analysis

**Figure 2:**
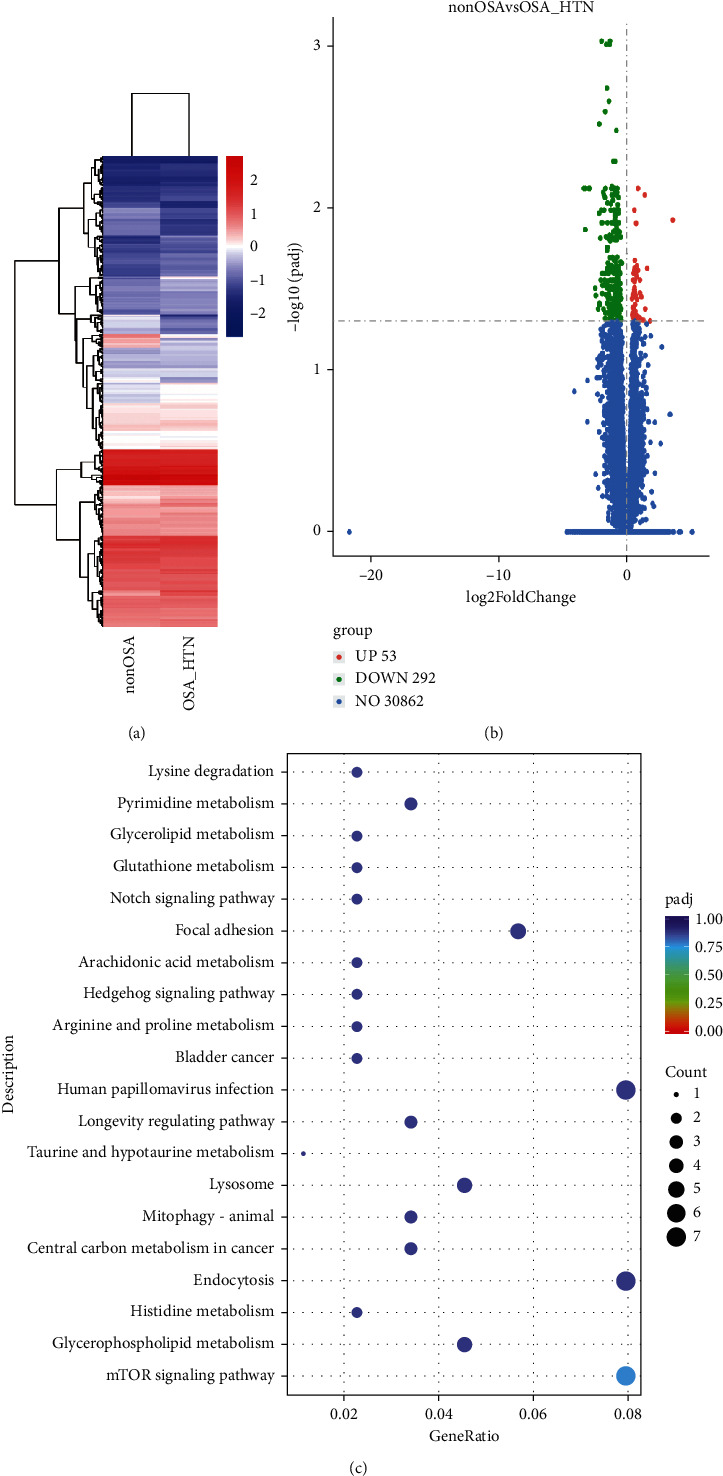
Profiles of differentially expressed gene transcripts between control patients without obstructive sleep apnea (non-OSA; apnea-hypopnea index (AHI) ≤5 without hypertension) and patients with OSA and hypertension (OSA-HTN; AHI ≥30). (a) Hierarchical heat maps, (b) volcano plots, and (c) top 20 overrepresented Kyoto Encyclopedia of Genes and Genomes pathways of the common differentially expressed genes. The colors (red, orange, green, light blue, and purple) represent the *Q* value (i.e., the lowest false discovery rate), whereas the variably sized dots represent the number of genes involved in each pathway.

The differentially expressed mRNAs between the OSA-HTN group and the non-OSA group that were previously selected were further screened by heat maps using unsupervised hierarchical cluster analysis (Figure 2(a)). Compared with those of the non-OSA group, the PBMC genes of the OSA-HTN group differed in that 53 genes showed upregulation, and 292 genes exhibited downregulation (Figure 2(b)). The top 10 differentially expressed mRNAs are listed in Table 3.

The results of the KEGG pathway analysis showed that the mTOR signaling pathway was enriched with the largest number of differential genes (*P*=0.006); the *Q* value was <0.05 (Figure 2(c)). We only listed the top 5 up- or downregulated genes of the relevant pathways, with the number of genes showing their corresponding gene IDs. On the other hand, in the mTOR signaling pathway, the WD repeat domain 24; disheveled segment polarity protein 1; HRas proto-oncogene, GTPase; Unc-51 like autophagy activating kinase 1; serine/threonine kinase 11 (STK11); and telomere maintenance 2 genes were downregulated in the OSA-HTN group, but not in the non-OSA group (Table 4).

## 4. Discussion

In this study, we found that the severe-OSA patients with hypertension and those without hypertension had obvious sleep architecture disturbances that manifested as increased stage N1 sleep as well as decreased stages N2 and N3 sleep accompanied by repeated arousals. Their gut microbiota's relative abundance decreased, especially SCFA-producing bacteria involving *Megamonas*, *Gemmiger*, *Dialister*, or *Oscillibacter*. The downregulation of arginine and proline metabolism was examined by functional analysis of microbiota. In addition, the mTOR signaling pathway was enriched with the largest number of differential genes from PBMCs.

### 4.1. OSA-Induced Gut Microbial Dysbiosis

OSA is a common sleep disorder that is characterized by repeated upper airway obstruction causing IH and SF. In patients with OSA, arousal is an important mechanism for reopening the upper airway and plays a crucial role in maintaining the upper airway in an open state when apnea occurs [2]. Moreover, it is an important factor in disruptions in the sleep architecture and gut microbiome [8, 14]. Because of the repeated hypoxia/reoxygenation periodic changes in the intestine, the appearance of a hypoxic environment in the intestine is conducive to anaerobic bacterial growth. An increase in the number of anaerobic bacteria can reduce the integrity of intestinal epithelial cell tight junctions by increasing intestinal mucosal permeability and bacterial translocation, which contribute to gut microbiota dysbiosis [12–14].

The intestine is the human body's largest mucosal immune defense line. Intestinal symbiotic microorganisms can have a role in host immune regulation, whereas SCFAs can regulate immune cells and play an important role in maintaining the balance of the intestinal immune microenvironment [31]. In this study, we found that the abundance of SCFA-producing bacteria decreased in the severe-OSA patients with hypertension and those without hypertension. SCFAs have a direct anti-inflammatory effect on the intestine, promote mucus synthesis, reduce bacterial translocation, maintain intestinal integrity, and reduce the host's intestinal inflammatory response [31]. Our previous study also observed a decrease in SCFA-producing bacteria and increased proinflammatory cytokines in OSA patients [17]. Increased levels of inflammatory factors in OSA are deemed to be potentially related to its pathophysiological processes and complications [5]. SCFAs can reduce the release of tumor necrosis factor (TNF)-*α*, interleukin (IL)-2, and IL-6 through the histone deacetylase inhibitory pathway via the G-protein-coupled receptor (GPR) 41 and GPR43 receptors [31]. These data suggest that the changes in SCFA-producing bacteria and cytokines in OSA may be related to its pathophysiological processes.

### 4.2. OSA-Induced Disruption of Sleep Architecture Impact on the Gut Microbiota and Metabolic Changes

Interestingly, studies have shown that changes in sleep rhythm can be attributed to changes in the intestinal microbiota and the body's metabolism. Metabolic changes induced by SF may be partly mediated by gut microbiotal taxa alteration [12, 32]. The structural components and metabolites of intestinal bacteria, such as bacterial peptides and lipopolysaccharides, can induce IL-1*β* or TNF-*α* production, which in turn can induce the NREM stage; on the other hand, cortisol can inhibit the synthesis of these cytokines, which have the same rhythm as cortisol [33]. Therefore, we hypothesized that OSA may affect the gut microbiome owing to changes in the sleep structure and that dysbiosis in the gut microbiota may also regulate the sleep structure through their metabolites. The gut microbiotal composition and activity are related to the development and function of the central nervous system. The bidirectional network between the brain and the intestine is the brain-gut axis. The gut microbiota are related to the hypothalamic-pituitary-adrenal axis; both systems affect the normal sleep architecture [18, 19, 34].

A reduction in deep sleep may be related to increased blood pressure and may increase refractory hypertension prevalence [9]. A study has shown that post-REM sleep deprivation led to vascular endothelial dysfunction and increased blood pressure in rats [10]. The arousal index is negatively correlated with the N3 stage, and sleep architecture disruptions in OSA patients may be related to frequent awakenings at night—that is, repeated awakenings lead to SF, increased light sleep times, and reduced deep sleep and REM sleep times. Reduced sleep in the N3 stage is considered to be an independent risk factor for hypertension as the risk of hypertension increases in patients with low-level stage N3 sleep [11]. Therefore, we infer that OSA manifests as frequent arousals, leading to SF and decreased deep sleep, which may play an important role in OSA-associated hypertension. However, the sleep architecture of the patients with OSA-HTN was similar to that of the patients with OSA only. There are little researches on sleep architecture disturbances of OSA patients with or without hypertension. Stages N2 sleep and arousal times were increased of OSA patients with hypertension than patients with hypertension [35]. Recently, Gürün Kaya et al. [36] found that only the arousal index was significantly higher in severe-OSA patients with hypertension than severe-OSA patients; there was no statistically significant difference in stages N1, N2, and N3 sleep.

### 4.3. The Effect of Metabolites of Gut Microbiota on Blood Pressure

Notably, functional analysis of the gut microbiota revealed that arginine metabolism in patients with OSA-HTN was significantly reduced. Arginine participates in the synthesis of nitric oxide (NO), which declines with increased blood pressure. Sleep deprivation during REM reduces the phosphorylation levels of endothelial NO synthase, NO, and cyclic guanosine monophosphate in the aorta, which impairs NO-mediated vasodilation as well as vasomotor dysfunction and affects the development of hypertension [10]. Moreover, a reduction in SCFA-producing bacteria is another cause of OSA-associated hypertension. The interaction between SCFAs and the GPR olfactory receptor 78 is believed to be involved in regulating blood pressure. SCFAs bind to olfactory receptor 78 on the renal vascular smooth muscle to regulate renin's release, which leads to blood pressure alteration [37].

### 4.4. OSA Alters Microbiome, Sleep Architecture, and mTOR Signaling Pathway to Develop Hypertension

Moreover, sleep deprivation attenuates the mTOR signaling pathway's expression, which can regulate central and peripheral circadian clock functions [27, 28]. mTOR may be linked to the intestinal microbiota. The relative abundance of such microbiota as *Oscillibacter* and *Ruminococcus* in mice fed with a high-fat diet was altered using resveratrol and rapamycin to inhibit the mTOR pathway [26]. Through changes in phosphatidylinositol 3-kinase/phosphorylated protein kinase B, the mTOR pathway can be adjusted to regulate the intestinal barrier function directly, thereby altering the gut microbiotal composition [20]. Inhibition of tuberous sclerosis 1 can overactivate the mTOR complex 1 pathway, thus inhibiting the differentiation of intestinal goblet cells and Paneth cells. These reductions can cause reductions in mucus and antimicrobial peptide production as well as subsequent damage to the intestinal barrier [21].

The activation of the mTOR signaling pathway is thought to be closely related to hypertension. Studies have confirmed that the protein kinase B/mTOR pathway plays an important role in developing cerebrovascular diseases [24]. SCFAs can bind to the GPR43 as well as promote the production of regenerating islet-derived protein III-*γ* and human *β*-defensins by activating the mTOR pathway of intestinal epithelial cells. Regenerating islet-derived protein III-*γ* is a new type of antibacterial peptide, whereas *β*-defensins are peptides with broad-spectrum antibacterial activity; their reduction is directly involved in regulating the gut microbiota [38]. Chronic IH, combined with a high-salt diet, reportedly induces hypertension in an animal model. *Lactobacillus rhamnosus* can regulate the levels of intestinal metabolites and CD4^+^ T-cell-induced inflammation through mTOR, thereby alleviating hypertension development and suggesting a correlation between the gut microbiome and mTOR immune balance in hypertension [23]. mTOR complex 1 is upregulated in PBMCs of obese patients with type 2 diabetes. It may increase the competitive inhibition of B-cell lymphoma 6 DNA binding elements by the signal transducer and activator of transcription 3 through phosphorylation of this protein, thereby weakening the inhibitory effect of B-cell lymphoma 6 on monocyte chemoattractant protein 1, promoting the production of inflammation, and contributing to the development of atherosclerosis [25]. We previously found that the levels of inflammatory cytokines are elevated in OSA patients [17]. In this study, we also found that the STK11 gene was downregulated. The STK11 gene negatively regulates the mTOR signaling pathway, which can inhibit the mTOR pathway when energy is lacking. Its downregulation may result in the activation of the mTOR pathway [39]. Therefore, our findings comprehensively suggest that the regulation of the mTOR signaling pathway is involved in developing OSA-associated hypertension through its interaction with the disturbance of the gut microbiome and sleep architecture.

### 4.5. Limitation

First, the main limitation of this study was the small sample size, and RNA-sequencing analysis was not performed in all subjects. Second, we did not comprehensively analyze levels of SCFAs in blood or stool. Third, sleep architecture disturbances in OSA patients with or without hypertension are worthy of further investigation. Future studies should be conducted with a larger sample size and metabolites measured to address this limitation as well as carry out the microbiota transplant experiment in an animal model to examine the underlying mechanism.

## 5. Conclusions

This study revealed differences in the gut microbiome of patients with severe-OSA-HTN. Disruptions in the sleep architecture and changes in the gut microbiota may interact in OSA-associated hypertension's physiopathology, and the mTOR signaling pathway may also be involved in this critical mechanism. Our findings shed new insight into the underlying pathogenesis of OSA-associated hypertension. Further large-scale prospective cohort studies are needed to confirm these findings.

## Figures and Tables

**Table 1:** Participant characteristics.

	Non-OSA	OSA	*P* value
	(*n* = 12)	(*n* = 60)	
Gender (male/female)	6/6	57/3	NA^a^
Age (years, mean ± SD)	38.25 ± 8.06	42.12 ± 7.63	0.117
Height (cm)	165.25 ± 8.41	168.28 ± 6.81	0.180
Weight (kg)	65.38 ± 8.96	81.11 ± 12.01	<**0.001**
Body mass index (kg m^−2^)	23.81 ± 2.59	28.49 ± 3.76	<**0.001**
Waist circumference (cm)	80.00 ± 6.73	97.32 ± 8.04	<**0.001**
Hip circumference (cm)	94.42 ± 7.03	100.89 ± 6.09	**0.002**

^a^N/A: not analyzed. Non-OSA: subjects with apnea-hypopnea index (AHI) ≤5 without hypertension; OSA: patients with severe OSA (AHI ≥30) with/without hypertension. The statistical analysis was performed with a *t*-test. *P* values < 0.05 were considered significant.

**Table 2:** Polysomnographic data analysis among non-OSA, OSA, and OSA-HTN.

	Non-OSA (*n* = 12)	OSA (*n* = 27)	OSA-HTN (*n* = 33)	*F* value	*P* value	Post-hoc test		
						*P* value		
						Non-OSA vs. OSA	Non-OSA vs. OSA-HTN	OSA vs. OSA-HTN
Total sleep time (min)	394.23 ± 79.11	409.43 ± 106.39	420.27 ± 89.03	0.330	0.720	NA^a^	NA	NA
N1 sleep stage (%)	26.85 ± 15.03	52.61 ± 18.04	49.03 ± 17.32	9.330	<**0.001**	<**0.001**	<**0.001**	0.476
N2 sleep stage (%)	34.68 ± 17.29	21.48 ± 12.09	25.11 ± 11.51	4.486	**0.015**	**0.004**	**0.030**	0.254
N3 sleep stage (%)	19.53 ± 6.34	8.65 ± 7.76	10.77 ± 8.49	8.035	**0.001**	<**0.001**	**0.002**	0.294
NREM sleep stage (%)	81.04 ± 10.72	82.78 ± 14.59	84.93 ± 9.32	0.657	0.522	NA	NA	NA
REM sleep stage (%)	18.96 ± 10.72	17.22 ± 14.59	15.07 ± 9.32	0.657	0.522	NA	NA	NA
Sleep efficiency (%)	72.55 ± 10.93	73.43 ± 17.61	75.25 ± 12.87	0.152	0.859	NA	NA	NA
Sleep latency (min)	29.41 ± 26.84	21.73 ± 29.01	28.26 ± 32.95	0.332	0.718	NA	NA	NA
Wake after sleep onset	118.03 ± 58.77	125.74 ± 84.63	111.3 ± 67.15	0.087	0.917	NA	NA	NA
Arousal index(events/h)	3.21 ± 1.42	3.56 ± 3.64	2.77 ± 2.16	0.322	0.726	NA	NA	NA
Arousal index in REM	35.15 ± 14.54	44.63 ± 21.41	45.95 ± 18.74	1.681	0.194	NA	NA	NA
Arousal index in NREM	33.83 ± 11.13	59.35 ± 16.69	65.52 ± 18.95	8.092	**0.001**	<**0.001**	<**0.001**	0.189
Total sleep arousal index	34.52 ± 11.18	59.19 ± 16.26	63.6 ± 17.65	14.302	<**0.001**	<**0.001**	<**0.001**	0.318
Apnea-hypopnea index (events/h)	1.90 ± 1.4	53.78 ± 17.90	58.99 ± 19.81	52.001	<**0.001**	<**0.001**	<**0.001**	0.447
Obstructive apnea index (events/h)	0.61 ± 0.75	29.29 ± 17.59	31.96 ± 22.37	12.944	<**0.001**	<**0.001**	<**0.001**	0.659
Central apnea index (events/h)	0.13 ± 0.19	3.06 ± 5.87	2.70 ± 3.51	2.084	0.132	NA	NA	NA
Mixed apnea index (events/h)	0.06 ± 0.12	4.30 ± 6.71	4.64 ± 6.05	2.991	0.057	NA	NA	NA
Hypopnea index (events/h)	1.10 ± 1.08	17.10 ± 11.47	19.69 ± 12.97	12.545	<**0.001**	<**0.001**	<**0.001**	0.566
Oxygen desaturation index (events/h)	1.52 ± 1.48	49.97 ± 18.95	55.6 ± 18.53	49.580	<**0.001**	<**0.001**	<**0.001**	0.443
Lowest oxygen saturation (%)	90.00 ± 4.11	72.22 ± 9.19	70.88 ± 9.43	25.112	<**0.001**	<**0.001**	<**0.001**	0.915
Average oxygen saturation (%)	95.92 ± 1.51	92.59 ± 2.94	91.48 ± 3.43	9.624	<**0.001**	**0.001**	**<0.001**	0.277
Mean heart rate	60.67 ± 8.91	67.22 ± 9.28	68.70 ± 12.1	2.494	0.090	NA	NA	NA
Arrhythmia index (events/h)	7.95 ± 16.36	17.89 ± 50.59	9.35 ± 25.60	0.618	0.542	NA	NA	NA
Maximum heart rate	96.67 ± 16.59	104.26 ± 13.97	101.91 ± 12.92	1.159	0.320	NA	NA	NA
Minimum heart rate	51.25 ± 7.66	53.22 ± 8.05	51.52 ± 7.80	0.453	0.637	NA	NA	NA
Blood pressure elevation index (events/h)	4.94 ± 5.19	29.61 ± 21.12	35.30 ± 26.19	8.405	**0.001**	**0.002**	**<0.001**	0.446
The highest systolic blood pressure (mmHg)	140.67 ± 23.60	185.48 ± 42.82	199.88 ± 37.85	11.020	**<0.001**	**0.001**	**<0.001**	0.287
Average systolic blood pressure (mmHg)	111.67 ± 16.29	119.85 ± 15.48	141.97 ± 21.97	15.339	**<0.001**	0.211	**<0.001**	**<0.001**
Average diastolic blood pressure (mmHg)	75.58 ± 10.39	82.78 ± 8.22	96.48 ± 12.57	20.529	**<0.001**	0.064	**<0.001**	**<0.001**

^a^Not analyzed. Non-OSA: apnea-hypopnea index (AHI) ≤5 without hypertension, OSA: severe OSA (AHI ≥30) without hypertension, and OSA-HTN: severe OSA with hypertension. *P* values < 0.05 were considered significant.

**Table 3:** Top 10 differentially expressed mRNAs in severe-OSA with HTN patients compared with non-OSA without hypertension subjects.

Upregulated mRNAs			Downregulated mRNAs		
Transcript ID	*P* value	log_2_FoldChange	Transcript ID	*P* value	log_2_FoldChange
(Ensembl_Gene_ID)			(Ensembl_Gene_ID)		
ENSG00000123505	1.09*E* − 0.5	0.871616484	ENSG00000268350	8.82*E* − 0.8	−1.315999106
ENSG00000242371	1.86*E* − 0.5	1.397821661	ENSG00000149591	1.17*E* − 0.7	−1.979312814
ENSG00000079335	3.60*E* − 0.5	0.585897618	ENSG00000142552	1.91*E* − 0.7	−1.614114348
ENSG00000196735	4.68*E* − 0.5	3.57106113	ENSG00000265982	2.45*E* − 0.7	−1.351438572
ENSG00000102189	5.90*E* − 0.5	0.722100597	ENSG00000260428	5.71*E* − 0.7	−1.560721207
ENSG00000087502	0.000142017	0.623605419	ENSG00000182154	8.22*E* − 0.7	−1.399962275
ENSG00000149308	0.000163471	0.779073878	ENSG00000160318	1.11*E* − 0.6	−1.698375943
ENSG00000197147	0.000177374	0.772560812	ENSG00000265206	1.52*E* − 0.6	−2.155095634
ENSG00000159256	0.000183054	0.612216032	ENSG00000175482	1.87*E* − 0.6	−0.821303269
ENSG00000134545	0.000188855	1.578550396	ENSG00000089820	3.27*E* − 0.6	−0.924629667

*P* values < 0.05 were considered significant.

**Table 4:** KEGG differential genes analysis in severe-OSA with HTN patients compared with non-OSA without hypertension subjects.

Expression	ID	Pathways	Counts	*P* value	Gene ID
Downregulation	hsa04150	mTOR signaling pathway	6	0.006583727	WDR24, DVL1, HRAS, ULK1, STK11, TELO2
	hsa00564	Glycerophospholipid metabolism	4	0.015867516	TAZ, PTDSS2, PNPLA6, DGKZ
	hsa05230	Central carbon metabolism in cancer	3	0.037543817	SIRT6, HRAS, G6PD
	hsa04137	Mitophagy-animal	3	0.040725158	RHOT2, HRAS, ULK1
	hsa05165	Human papillomavirus infection	7	0.052551422	TCIRG1, SCRIB, HDAC10, RFNG, DVL1, HRAS, LAMC3

Upregulation	hsa04727	GABAergic synapse	2	0.013274614	SLC38A2, TRAK2
	hsa03018	RNA degradation	2	0.014053501	SKIV2L2, TTC37
	hsa05332	Graft-versus-host disease	1	0.042416288	KLRC1
	hsa00340	Histidine metabolism	1	0.057882946	CARNMT1
	hsa02010	ABC transporters	1	0.100443008	ABCD2

*P* values < 0.05 were considered significant.

## Data Availability

All data generated or analyzed during this study are included in this article.

## Abbreviations

OSA: Obstructive sleep apnea
mTOR: Mechanistic target of rapamycin
16S rRNA: 16S ribosomal RNA
IH: Intermittent hypoxia
SF: Sleep fragmentation
CPAP: Continuous positive airway pressure
OSA-HTN: OSA and hypertension
REM: Rapid eye movement
NREM: Non-REM
SCFAs: Short-chain fatty acid
PSG: Polysomnography
PBMCs: Peripheral blood mononuclear cells
SBP: Systolic blood pressure
DBP: Diastolic blood pressure
KEGG: Kyoto Encyclopedia of Genes and Genomes
ANOVA: One-way analysis of variance
STK11: Serine/threonine kinase 11
D: Standard deviation
TNF: Tumor necrosis factor
IL: Interleukin
GPR: G-protein-coupled receptor
NO: Nitric oxide.

## Supplementary Materials

Supplementary Materials Supplementary Figure 1: Characteristics of sequencing data in the operational taxonomic units (OTUs) (A), the mean community diversity indices [Chao (B), Shannon (C), and Simpson (D)]. Non-OSA: apnea-hypopnea index (AHI)≤5 without hypertension, OSA: severe-OSA (AHI ≥ 30) without hypertension, and OSA-HTN: severe OSA with hypertension.
