# Supplementary figures and images for: Disturbances of the Gut Microbiota, Sleep Architecture, and mTOR Signaling Pathway in Patients with Severe Obstructive Sleep Apnea-Associated Hypertension
Source: Int J Hypertens. 2021 Nov 30;2021:9877053. doi: 10.1155/2021/9877053 (PMC8651365; doi:10.1155/2021/9877053)

Supplementary Figure 1S

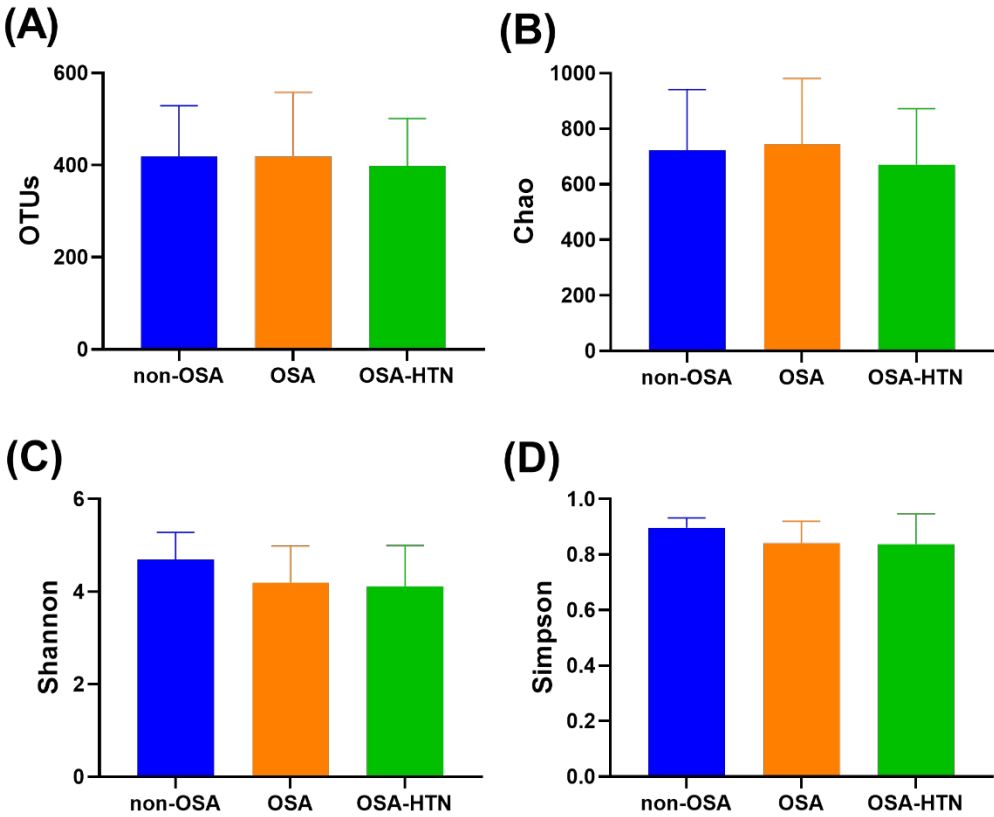

Supplement: Supplementary Materials — Supplementary Figure 1: Characteristics of sequencing data in the operational taxonomic units (OTUs) (A), the mean community diversity indices [Chao (B), Shannon (C), and Simpson (D)]. Non-OSA: apnea-hypopnea index (AHI)≤5 without hypertension, OSA: severe-OSA (AHI ≥ 30) without hypertension, and OSA-HTN: severe OSA with hypertension. [file 9877053.f1.pdf]
